# Comparing the intra- and inter-rater reliability of two outcomes, two software platforms, and three levels of surgical experience for radiographic measurements of surgically treated osteoporotic vertebral compression fractures

**DOI:** 10.1016/j.bas.2026.106070

**Published:** 2026-04-27

**Authors:** Andreas Tsoupras, Anne Tabard-Fougère, Khalid Al Taha, Pietro Feltri, Sana Boudabbous, Dennis E. Dominguez, Nicolas Lauper

**Affiliations:** a Spine Team, Division of Orthopaedics Surgery and Musculoskeletal Trauma Care, Geneva University Hospitals and the University of Geneva's Faculty of Medicine, Geneva, Switzerland; b Department of Neurosurgery, Valais Hospital, Sion, Switzerland; c Musculoskeletal Unit, Division of Radiology, Geneva University Hospitals and the University of Geneva's Faculty of Medicine, Geneva, Switzerland

**Keywords:** Osteoporotic vertebral compression fracture, Standing X-ray, Intra-rater reliability, Inter-rater reliability, Inter-software agreement, Responsiveness, Kyphotic deformity

## Abstract

**Introduction:**

Osteoporotic vertebral compression fractures (OVCFs) present diagnostic challenges, especially in elderly patients with low bone density. Radiographic measurement is key to surgical decision-making, but their reliability in these patients with lower radiological density is questionable.

**Research question:**

What is the intra- and inter-rater reliability and responsiveness to the measurement of two commonly used radiographic parameters—vertebral kyphotic angle (VKA) and vertebral body height (VBH)—using two different DICOM software platforms for patients undergoing surgical treatment for OVCFs?

**Material and methods:**

Patients treated surgically for OVCFs were randomly extracted. Sagittal vertebral body deformity was evaluated using the VKA and the VBH (pre-operative, post-operative and change over time). One operator repeated measurements with one-month interval (intra-rater reliability), using two software platforms (OsiriX MD and Weasis). Three randomly assigned surgeons per platform performed blindly measurements (inter-rater). Reliability was assessed using intraclass correlation coefficients (ICC).

**Results:**

Thirty-four patients (71% females; mean age 75.3 ± 9.4 years) were included. Intra-rater and inter-rater reliability were reported as good-to-excellent (ICC: 0.75-0.90) for VKA, and good-to-moderate (ICC: 0.50-0.75) for VBH. Reliability for measuring post-operative change was moderate for both outcomes. Both software platforms perform comparably with good agreement.

**Discussion and conclusions:**

This was the first study to assess the reliability and responsiveness of postoperative radiographic changes in OVCF surgery across two different software platforms. VKA is a more reliable measurement than VBH. These findings support using VKA to guide treatment and monitoring recovery, although measurement error remain important considerations in elderly patients with low bone density.

## Highlights

• VKA demonstrated excellent reliability for pre-/post-op assessment vs moderate reliability for VBH measurements.
• Post-surgery deformity (responsiveness) showed only moderate inter-rater agreement for both VKA and VBH.
• OsiriX MD and Weasis showed no significant difference in results, making them interchangeable for these measurements.
• For osteoporotic vertebral compression fractures, VKA is a more trustworthy radiographic parameter than VBH.

## 1 Introduction

The number of osteoporotic vertebral compression fractures (OVCFs) diagnosed in recent decades has increased considerably due to general population aging, and spine surgeons must regularly deal with them in their daily practice (Al et al., 2024). Proper characterization is essential for guiding treatment, and several classifications have been developed to help clinicians’ decision-making processes; however, to date, no universal consensus exists (Parreira et al., 2017; Scherer et al., 2024). Moreover, several methods exist for measuring the local deformities caused by compression fractures, including the vertebral kyphotic angle (VKA) and loss of vertebral body height (VBH) (Sadiqi et al., 2017; Keynan et al., 2006). Accurate quantification of kyphotic angle enables clinicians to monitor segmental and global sagittal alignment, which is critical for predicting functional impairment, pain severity, and the likelihood of further vertebral collapse or non-union(Chen et al., 2024; Oishi et al., 2025; Ensrud et al., 1997)Goldstein and Cnctord, 2015, Keynan et al., 2006, Mokkink et al., 2021(Chen et al., 2024; Oishi et al., 2025; Ensrud et al., 1997). Kyphotic angle measurement is a key radiological parameter in the OF classification system, which integrates both radiographic and clinical parameters, including VKA and VBH, to categorize fracture severity and guide treatment decisions, particularly in surgical candidates (Al et al., 2024; Schönrogge et al., 2022; Rajasekaran et al., 2017). Although 3D imaging using CT scans and/or MRI is now part of the standard diagnostic workup, a conventional standing X-ray assessment remains the keystone of therapeutic decision-making (Al et al., 2024). Both radiologists and spine specialists require precise diagnostic imaging. This becomes challenging when older adult patients have demineralized bones of lower radiological density (Schröder et al., 2024), which can lead to inter-rater variability.

Measurement errors are usually associated with the observer. However, the software used for radiographic analysis may also lead to measurement differences. No studies to date have evaluated the reliability or agreement between the measurements obtained using different software platforms for subsequent surgical planning and follow-up.

Using a reliable, reproducible measurement method is essential to establishing shared diagnostic frameworks between radiologists and clinicians, thereby ensuring consistent, coordinated decision-making. Of the nine measurement properties used to evaluate the quality of health-related patient-reported outcomes, intra- and inter-rater reliability and responsiveness (longitudinal validity) are especially important when patients are monitored over time (Mokkink et al., 2021; Mokkink TCKDSPAJPD et al., 2010; Terwee PJCRLJTBCD et al., 2021).

In this context, the present study aimed to assess the intra- and inter-rater reliability and the responsiveness of two commonly used radiographic parameters, VKA and VBH, measured in patients undergoing surgical treatment for OVCFs. We compared three levels of surgical experience and used two different DICOM software platforms.

## 2 Materials and methods

### 2.1 Study design

This cross-sectional reliability and methodological study evaluated the intra- and inter-rater reliability of radiographic measurements taken in patients treated surgically for OVCFs. The study incorporated three levels of surgical expertise, a longitudinal responsiveness assessment (change in postoperative measurements), and a software platform comparison between OsiriX MD and Weasis.

### 2.2 Participants

Patients were randomly selected retrospectively from a database of patients who had been treated surgically for osteoporotic fractures at Geneva University Hospitals (HUG) from 2018 to 2024. The local ethics committee approved this study (CCER no. 2023-01942) as all the subjects had provided a written informed consent form permitting the use of their data in research projects.

All the patients had undergone biplanar, low-dose, whole-spine X-rays in the standing position before surgery, in the supine position immediately after surgery, and in the standing position again 2–5 days later.

Patient inclusion criteria were 1) presenting with an OVCF (classified using the OF system) (Al et al., 2024; Schönrogge et al., 2022; Rajasekaran et al., 2017) that underwent surgical treatment by kyphoplasty alone or kyphoplasty associated with posterior instrumentation; 2) being aged >18 years old; and 3) having available preoperative and postoperative radiographic assessment records. Exclusion criteria were: 1) refusing to participate; 2) incomplete data; and 3) presenting with other pathological fractures due to oncological or infectious diseases or a history of previous spinal surgery with or without surgical implants.

The sample size was defined using the ICC Sample Size package in R software (v 1.0) (R-Core-Team, 2020) (Borg and BAOJSKL, 2022), where intra-class correlation (ICC) was defined as the primary outcome. The sample size of 33 patients and a significance level of 0.05 gave a power of 80% and an anticipated ICC of 0.80. There were three raters per type of software. Uniform distributions were applied to the subgroups of patients extracted from the database based on patients’ OF classification stage (Parreira et al., 2017; Scherer et al., 2024).

### 2.3 Study procedure

Each patient recruited underwent a low-dose biplanar X-ray evaluation using the EOS® System (Biospace Med, Paris, France) as part of their clinical consultation. Each patient recruited had undergone three radiological evaluations: 1) pre-operatively in the standing position; 2) immediately post-operatively in the supine position; and 3) 2–5 days post-operatively in the standing position. Each patient had undergone a vertebral augmentation using the SpineJack device (Stryker, MI, USA).

### 2.4 Imaging analysis

Radiological data were extracted from hospital's Picture Archiving and Communication Systems (PACS). The WAESIS DICOM medical viewer (software #1) and OsiriX MD software (Pixmeo SARL, CH-1233 Bernex, Switzerland) (software #2) were used for our radiological analyses.

Four raters (one orthopedic surgery resident, two senior spine surgeons, and one physician) independently measured the radiographs. All the raters were blinded to earlier measurements.

### 2.5 Primary outcomes

**Fig. 1:**
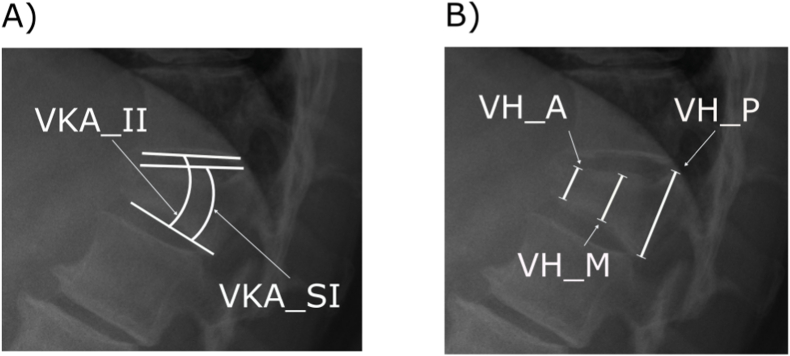
Outcomes illustration. A) The VKA evaluated using the Cobb angle measurement between the superior and inferior endplates of the index vertebra (VKA_SI) and between the inferior endplate of the index vertebra and the vertebra above it (VKA_II); B) The VBH evaluated at the anterior (VBH_A), medial (VBH_M), and posterior (VBH_P) parts of the vertebra.

Each patient's vertebral deformity was evaluated using the two most commonly used measurements described in literature and used in clinical practice: 1) the VKA, using the Cobb angle measurement between the superior and inferior endplates of the index vertebra (VKA_SI) and between the inferior endplate of the index vertebra and the vertebra above (VKA_II) (Fig. 1-A); and 2) the VH evaluated at the vertebra's anterior (VBH_A), medial (VBH_M), and posterior (VBH_P) part of the vertebra (Fig. 1-B). The ratio based on the measurement of the posterior part of the vertebra was also computed as both VBH_AP= VBH_A/VBH_P and VBH_MP= VBH_M/VBH_P.

### 2.6 Secondary outcomes

The demographic parameters used to describe patient characteristics were age, gender, and BMI. The T-scores from dual-energy X-ray absorptiometry (DEXA) were measured to describe patients’ bone density: ≥ −1.0 was normal, from −1.0 to −2.5 was considered low bone density, and ≤ −2.5 signified osteoporosis.

### 2.7 Statistical analysis

All statistical analyses were done using R software (version 4.4.1, R Development Core Team, Vienna, Austria, 2024) and the RStudio interface (version 2024.09.0, Posit Software, PBC). The level of significance was set at *p* < 0.05.

Our descriptive analyses reported a mean (plus standard deviation) for continuous outcomes and numbers (plus %) for categorical outcomes. Comparisons were made using paired Student's t-tests, and results were reported as a mean (standard deviation).

To evaluate intra-rater reliability, a single rater (ATF) measured the radiographs twice, using both software platforms, with a one-month interval. The mean difference between the evaluations made at J0 and J1 (1 month interval) were reported for radiographic outcomes.

To evaluate the inter-rater reliability, radiographs were measured blind using both software platforms by one orthopedic surgery resident (AT/KAT), one senior spine surgeon (DD/NL), and one physician (ATF). The mean inter-rater difference was reported for each outcome.

The ICC estimates, the standard error of measurement (SEM), and the smallest detectable change (SDC), each with their 95% confidence intervals (95%CI), were calculated using the *irr* package in R based on single-rating, absolute agreement, two-way mixed-effects.

Agreement between the two software platforms was evaluated using the two-ICC and Bland–Altman plots with their respective limits of agreement (±1.96 SD).

The criteria for interpreting the ICC were defined as follows: ICC ≥0.90 = excellent, ICC from 0.75 to 0.90 = good, ICC from 0.50 to 0.75 = moderate, and ICC <0.50 = is poor (Shrout and Fleiss, 1979).

## 3 Results

### 3.1 Participants

This study retrospectively included 34 consecutive patients who consulted at our institution between 2018 and 2024. Patients’ baseline characteristics are reported in Table 1. There were 24 females (71%), overall mean age was 75.3 (9.4) years old a mean BMI of 26.3 (4.3).

**Table 1:** Baseline population characteristics of the included patients surgically treated osteoporotic vertebral compression fractures (n = 34).

	Value (n = 34)
**Demographic characteristics**	
Female, n (%)	24 (71%)
Mean age (SD), years	75.3 (±9.4)
Mean BMI (SD), kg/m^2^	26.3 (±4.3)
**Fracture characteristics**	
T-score	
> −2.5, n (%)	24 (71%)
≤ −2.5, n (%)	10 (29%)
Fracture location	
Mid-thoracic T6–T10, n (%)	2 (6%)
Thoracolumbar junction T11–L2, n (%)	29 (85%)
Lumbar L2–L4, n (%)	3 (9%)
OF classification	
OF 2, n (%)	9 (26%)
OF 3, n (%)	9 (26%)
OF 4, n (%)	7 (22%)
OF 5, n (%)	9 (26%)

BMI = body mass index; n = number; T = thoracic; L = lumbar; OF = osteoporotic fracture.

### 3.2 Change after surgery

**Fig. 2:**
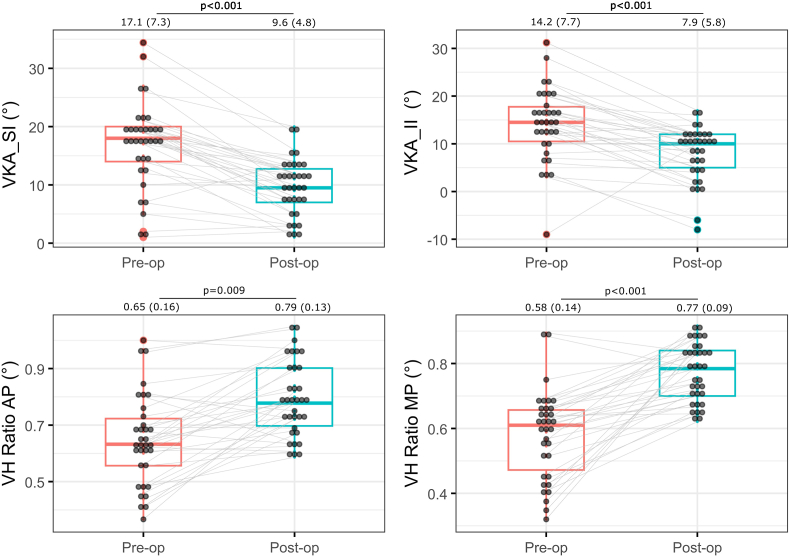
Postoperative changes in the vertebral body measurements of 34 patients. *Comparisons were made using paired Student's t-tests and results were reported as a mean (standard deviation). VKA_SI = Cobb angle measurement between the superior and inferior endplates of the index vertebra; VKA_II = Cobb angle measurement between the* inferior endplate of the index vertebra and the vertebra above it*; VBH is vertebral body height ratio between the anterior and the posterior part of the vertebra (AP), and between the medial and the posterior (MP) part of the vertebra.*

As Fig. 2 illustrates, the measurements from the vertebral body radiographs performed pre- and post-operatively highlighted a significant mean restoration of both the VKA_II (95%CI: −9.4 to −5.6; p < 0.001; effect size [ES] = 1.23) and the VKA_SI (95%CI: −8.7 to −3.4; p < 0.001; ES = 0.94). Similarly, there was a significant mean restoration of both the VBH_AP ratio (95%CI: 0.09 to 0.20; p < 0.001; ES = 0.97) and the VBH_MP ratio (95%CI: 0.14 to 0.24; p < 0.001; ES = 1.64).

### 3.3 Intra-rater reliability

**Fig. 3:**
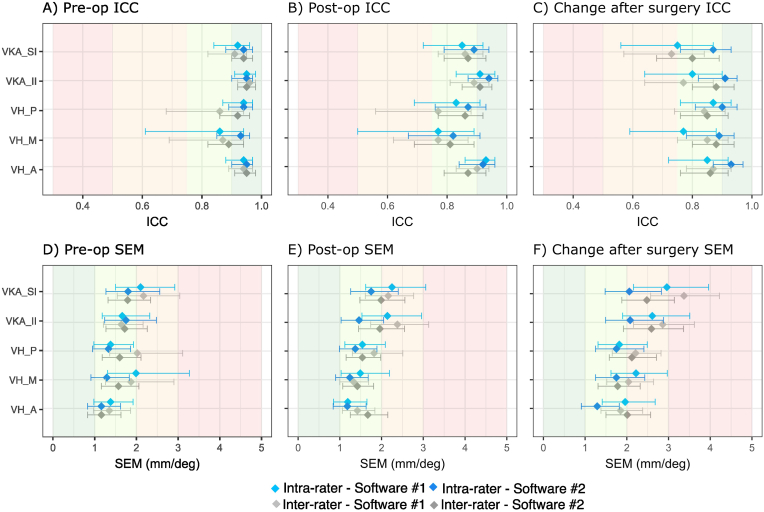
Intra- and inter-rater reliability. A/D) Pre-operative measurements. B/E) Post-operative measurements. C/F) Change after surgery measurements. *ICC = intraclass correlation coefficient; SEM = standard error measurement.*

Intra-rater reliability was excellent for every preoperative outcome (ICC >0.90) except the VBH_M when evaluated using software #1 (ICC = 0.86). For every preoperative outcome, SEMs ranged from 12 to 2.1 and SDCs ranged from 3.2 to 5.7 (Table 2 and Fig. 3 A**/D in blue**).

**Table 2:** Intra-rater reliability for pre-op, post-op, and change after surgery measurements.

	Mean (SD) Day#1	Mean (SD) Day#2	ICC (95%CI)	SEM (95%CI)	SDC (95%CI)
**Software #1**					
Preoperative outcomes					
VKA_SI	17.2 (7.3)	17.3 (7.6)	0.93 (0.86; 0.96)	2.1 (1.5; 2.9)	5.7 (4.1; 7.9)
VKA_II	14.4 (7.6)	14.9 (7.5)	0.95 (0.91; 0.98)	1.7 (1.2; 2.3)	4.6 (3.3; 6.4)
VBH_A	17.7 (5.6)	17.5 (5.2)	0.94 (0.88; 0.97)	1.4 (1.0; 1.9)	3.8 (2.7; 0.5.4)
VBH_M	17.8 (5.3)	16.4 (4.4)	0.86 (0.61; 0.94)	2.0 (1.3; 3.3)	5.5 (3.7; 9.1)
VBH_P	28.2 (5.4)	27.7 (5.1)	0.94 (0.87; 0.97)	1.4 (1.0; 1.9)	3.8 (2.7; 5.4)
Postoperative outcomes					
VKA_SI	9.3 (5.9)	9.8 (5.1)	0.86 (0.73; 0.92)	2.3 (1.6; 3.1)	6.3 (4.5; 8.4)
VKA_II	7.1 (7.2)	7.0 (6.6)	0.91 (0.83; 0.96)	2.1 (1.5; 3.0)	5.9 (4.2; 8.2)
VBH_A	20.4 (4.4)	20.2 (4.2)	0.93 (0.86; 0.96)	1.2 (0.9; 1.7)	3.3 (2.4; 4.5)
VBH_M	20.3 (2.7)	19.3 (3.1)	**0.77 (0.50; 0.89)**	1.5 (1.0; 2.2)	4.1 (4.0; 6.1)
VBH_P	25.4 (3.8)	25.1 (3.5)	0.83 (0.69; 0.91)	1.5 (1.1; 2.1)	4.3 (3.1; 5.8)
Change after surgery outcomes					
VKA_SI	7.9 (6.0)	7.5 (5.8)	**0.76 (0.57; 0.87)**	3.0 (2.2; 4.0)	**8.3 (6.1; 11)**
VKA_II	7.3 (5.9)	8.0 (5.5)	0.80 (0.64; 0.90)	2.6 (1.9; 3.5)	**7.2 (5.2; 9.8)**
VBH_A	2.7 (4.9)	2.7 (5.1)	0.85 (0.72; 0.92)	2.0 (1.4; 2.7)	5.4 (4.0; 7.5)
VBH_M	2.5 (4.6)	2.9 (4.5)	**0.77 (0.59; 0.88)**	2.2 (1.6; 3.0)	**6.2 (4.5; 8.2)**
**Software #2**					
Preoperative outcomes					
VKA_SI	17.1 (7.3)	16.2 (6.7)	0.94 (0.88; 0.97)	1.8 (1.3; 2.6)	4.9 (3.5; 7.1)
VKA_II	14.2 (7.7)	13.4 (7.1)	0.95 (0.90; 0.97)	1.8 (1.2; 2.5)	4.9 (3.4; 6.9)
VBH_A	18.1 (5.2)	17.9 (5.2)	0.95 (0.90; 0.97)	1.2 (0.8; 1.6)	3.2 (2.3; 4.5)
VBH_M	16.3 (4.8)	16.9 (4.5)	0.93 (0.86; 0.97)	1.3 (0.9; 1.8)	3.6 (2.5; 5.1)
VBH_P	28.0 (5.6)	27.6 (4.9)	0.94 (0.89; 0.97)	1.3 (1.0; 1.9)	3.7 (2.7; 5.1)
Postoperative outcomes					
VKA_SI	9.5 (5.1)	8.9 (5.4)	0.90 (0.81; 0.95)	1.7 (1.3; 2.4)	4.8 (3.5; 6.6)
VKA_II	7.3 (6.5)	6.9 (5.7)	0.95 (0.90; 0.97)	1.5 (1.1; 2.1)	4.1 (3.0; 5.7)
VBH_A	20.4 (4.1)	20.8 (4.1)	0.92 (0.84; 0.96)	1.2 (0.8; 1.6)	3.8 (2.4; 4.5)
VBH_M	19.9 (2.9)	19.8 (2.9)	0.82 (0.67; 0.91)	1.2 (0.9; 1.7)	3.4 (2.5; 4.7)
VBH_P	25.9 (3.9)	25.8 (3.4)	0.87 (0.76; 0.93)	1.4 (1.0; 1.9)	3.3 (2.7; 5.2)
Change after surgery outcomes					
VKA_SI	7.6 (5.5)	7.3 (5.8)	0.87 (0.76; 0.93)	2.1 (1.5; 2.8)	5.8 (4.1; 7.9)
VKA_II	6.9 (5.2)	6.5 (5.2)	0.85 (0.72; 0.92)	2.0 (1.5; 2.8)	5.6 (4.1; 7.6)
VBH_A	2.4 (5.0)	2.9 (4.6)	0.93 (0.87; 0.97)	1.3 (0.9; 1.8)	4.9 (2.5; 5.1)
VBH_M	3.6 (5.2)	2.9 (4.7)	0.89 (0.78; 0.94)	1.8 (1.3; 2.4)	3.6 (3.5; 6.8)

SD = standard deviation; ICC = intraclass correlation coefficient; SEM = standard error measurement; VKA = vertebral kyphotic angle; VBH = vertebral body height. Values in **bold** are outcomes with lowest reliability (**ICC<0.80**).

Intra-rater reliability for postoperative outcomes was good to excellent (ICC >0.75), with SEMs ranging from 1.2 to 2.3 and SDCs ranging from 2.4 to 6.2. The lowest value (ICC = 0.77) was reported for VBH_M evaluated using software #1 (Table 2 and Fig. 3 B**/E in blue**).

Intra-rater reliability was good to moderate when evaluating change after surgery using software #1 (ICC >0.75), and SEMs ranged from 2.0 to 3.0 and SDCs ranged from 5.5 to 8.3 (Table 2 and Fig. 3 C**/F in blue**), with the lowest ICC values being for the VKA_SI (ICC = 0.76) and VBH_M (ICC = 0.77). Intra-rater reliability was excellent (ICC >0.85) for every outcome when evaluating the change after surgery using software #2, with SEMs ranging from 1.3 to 2.1 and SDCs ranging from 3.5 to 5.8.

### 3.4 Inter-rater reliability

Inter-rater reliability was excellent for every preoperative outcome (ICC >0.90) except the VBH_P (ICC = 0.86) and the VBH_M (ICC = 0.87) evaluated using software #1. SEMs for every preoperative outcome ranged from 1.2 to 2.2 and SDCs ranged from 1.6 to 4.3 (Table 3 and Fig. 3 A**/D in grey**).

**Table 3:** Inter-rater reliability for pre-op, post-op, and change after surgery measurements.

	Mean (SD) Op#1	Mean (SD) Op#2	Mean (SD) Op#3	ICC (95%CI)	SEM (95%CI)	SDC (95%CI)
**Software #1**						
Preoperative outcomes						
VKA_SI	17.3 (7.3)	16.2 (6.4)	18.2 (7.2)	0.91 (0.83; 0.95)	2.2 (1.6; 3.1)	6.1 (4.4; 8.5)
VKA_II	14.4 (7.6)	14.2 (7.3)	14.3 (7.8)	0.96 (0.92; 0.98)	1.6 (1.2; 2.2)	4.6 (3.4; 5.9)
VBH_A	17.7 (5.6)	18.7 (5.5)	17.7 (5.3)	0.94 (0.89; 0.97)	1.4 (1.0; 1.9)	3.8 (2.7; 5.2)
VBH_M	17.8 (5.3)	18.0 (5.2)	15.9 (4.7)	0.87 (0.69; 0.94)	1.9 (1.3; 2.9)	5.2 (3.5; 8.1)
VBH_P	28.2 (5.4)	27.5 (5.5)	30.0 (5.2)	0.86 (0.68; 0.94)	2.0 (1.4; 3.1)	5.6 (3.8; 8.6)
Postoperative outcomes						
VKA_SI	9.3 (5.9)	9.5 (6.0)	9.6 (5.6)	0.87 (0.78; 0.92)	2.2 (1.7; 2.9)	6.2 (4.5; 7.9)
VKA_II	7.1 (7.2)	7.9 (6.6)	6.1 (6.9)	0.89 (0.81; 0.94)	2.4 (1.7; 3.1)	6.6 (4.8; 8.6)
VBH_A	20.4 (4.4)	20.8 (4.0)	20.6 (4.1)	0.90 (0.83; 0.94)	1.4 (1.1; 1.8)	3.9 (3.0; 5.1)
VBH_M	20.3 (2.7)	20.2 (2.7)	19.4 (2.7)	**0.77 (0.62; 0.87)**	1.3 (1.0; 1.7)	3.7 (2.8; 4.7)
VBH_P	25.4 (3.8)	24.6 (3.4)	26.6 (3.4)	**0.77 (0.56; 0.88)**	1.8 (1.3; 2.5)	5.0 (3.5; 6.9)
Change after surgery outcomes						
VKA_SI	7.8 (6.1)	6.7 (6.3)	8.6 (5.8)	**0.72 (0.57; 0.84)**	3.3 (2.5; 4.1)	**9.2 (7; 11.5)**
VKA_II	7.3 (5.9)	6.3 (5.6)	8.1 (6.0)	**0.77 (0.64; 0.87)**	2.9 (2.2; 3.6)	**7.9 (5.9; 10)**
VBH_A	2.7 (4.9)	2.1 (4.6)	3.0 (5.1)	0.87 (0.78; 0.93)	1.9 (1.4; 2.4)	5.1 (3.8; 6.6)
VBH_M	2.5 (4.6)	2.2 (5.3)	3.5 (5.3)	0.85 (0.75; 0.92)	2.0 (1.5; 2.6)	5.7 (4.2; 7.4)
**Software #2**						
Preoperative outcomes						
VKA_SI	17.1 (7.5)	17.6 (6.9)	17.0 (6.7)	0.94 (0.90; 0.97)	1.8 (1.4; 2.4)	5.1 (3.8; 6.6)
VKA_II	14.2 (7.7)	14.3 (8.1)	13.6 (7.5)	0.95 (0.92; 0.98)	1.7 (1.3; 2.3)	4.8 (3.5; 6.2)
VBH_A	18.1 (5.2)	17.1 (5.3)	17.2 (5.0)	0.95 (0.91; 0.98)	1.2 (0.8; 1.6)	3.2 (2.3; 4.5)
VBH_M	16.3 (4.8)	17.4 (4.3)	16.7 (4.7)	0.89 (0.82; 0.94)	1.6 (1.2; 2.1)	4.4 (3.3; 5.8)
VBH_P	28.0 (5.6)	29.0 (4.9)	28.6 (4.7)	0.92 (0.86; 0.96)	1.6 (1.2; 2.1)	4.4 (3.3; 5.9)
Postoperative outcomes						
VKA_SI	9.5 (5.1)	9.2 (5.7)	9.7 (5.7)	0.88 (0.79; 0.93)	2.0 (1.5; 2.6)	5.5 (4.1; 7.1)
VKA_II	7.3 (6.5)	6.3 (7.1)	7.0 (7.2)	0.93 (0.87; 0.96)	2.0 (1.4; 2.6)	5.4 (4.0; 7.1)
VBH_A	20.4 (4.1)	19.7 (4.7)	20.0 (4.7)	0.87 (0.79; 0.93)	1.7 (1.3; 2.2)	4.6 (3.4; 5.9)
VBH_M	19.9 (2.9)	19.8 (3.2)	19.7 (3.3)	0.81 (0.69; 0.89)	1.1 (1.1; 1.8)	3.9 (3.0; 4.9)
VBH_P	25.9 (3.9)	26.0 (4.1)	26.5 (3.7)	0.86 (0.77; 0.92)	1.5 (1.2; 2.0)	4.3 (3.3; 5.5)
Change after surgery outcomes						
VKA_SI	7.6 (5.5)	8.4 (5.4)	7.3 (5.4)	**0.79 (0.66; 0.89)**	2.5 (1.9; 3.2)	**6.9 (5.2; 8.8)**
VKA_II	6.9 (5.2)	8.0 (6.1)	6.6 (6.3)	**0.82 (0.71; 0.90)**	**2.7 (2.0; 3.4)**	**7.4 (5.5; 9.3)**
VBH_A	2.4 (5.0)	2.7 (5.3)	2.8 (5.2)	0.86 (0.76; 0.92)	2.0 (1.5; 2.6)	5.6 (4.1; 7.1)
VBH_M	3.6 (5.2)	2.4 (5.2)	3.0 (4.6)	0.88 (0.80; 0.94)	1.8 (1.3; 2.3)	4.9 (3.7; 6.5)

SD = standard deviation; ICC = intraclass correlation coefficient; SEM = standard error measurement; VKA = vertebral kyphotic angle; VBH = vertebral body height. Values in **bold** are outcomes with lowest reliability (**ICC<0.80**).

For the postoperative outcomes, inter-rater reliability was only excellent for the VKA_II (software #2) and the VBH_A (software #1) (ICC >0.90). It was good (ICC >0.75) for all the other postoperative outcomes, with ICCs >0.85, except for the medial and posterior VBH. For every postoperative outcome, SEMs ranged from 1.1 to 2.4 (Table 3 and Fig. 3 B**/E in grey**).

Inter-rater reliability was good to excellent (ICC >0.75) for every outcome when evaluating changes after surgery using software #2 (except VBH_P: ICC = 0.70), with SEMs ranging from 1.7 to 2.6. However, inter-rater reliability was moderate (ICC <0.75) for evaluating change after surgery using the software #1, with the lowest ICC value (0.63) being for the medial VBH, and the SEMs ranging from 1.7 to 3.4 (Table 3 and Fig. 3 C**/F in grey**).

### 3.5 Inter-software agreement

**Fig. 4:**
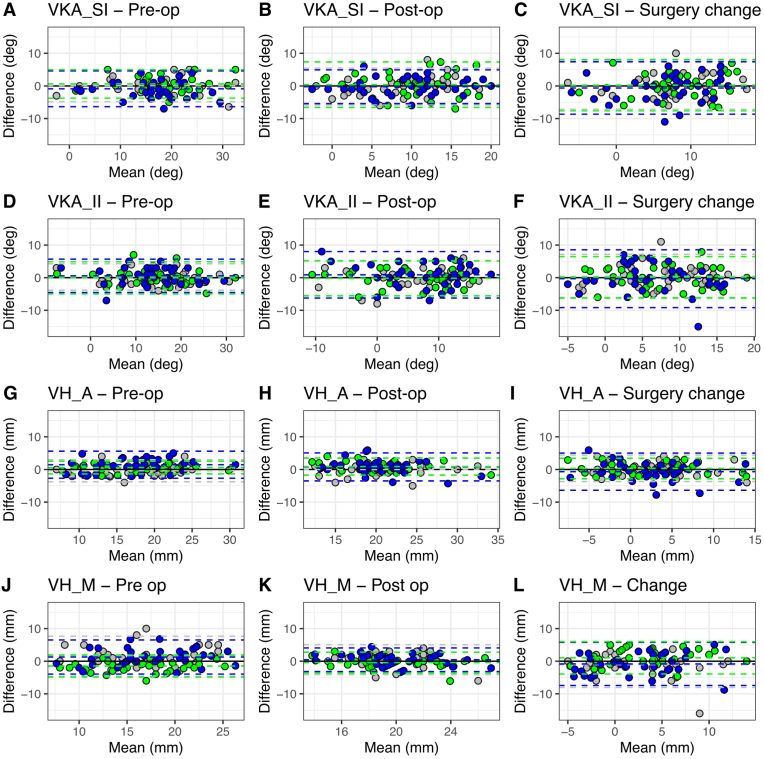
Inter-software agreement.

As Fig. 4 illustrates, inter-software agreement was not influenced by surgeons’ levels of experience. For preoperative VKA, limits of agreements ranged from −6.5 to 5.7°, with values higher than 5.0° only estimated by junior orthopedic surgeons. Similarly, all the preoperative VBH_A limits of agreements were <5.0°, except those of the junior orthopedic surgeons (from −2.9 to 5.6°). Preoperative VBH_M limits of agreements were only <5° for the senior orthopedic surgeons (−4.9 to 1.9°).

Limits of agreement for postoperative outcomes were >5° for the VKA angles (from −6.6 to 8.0) and around 5.0° for the VBH_A and VBH_M.

## 4 Discussion

The present study's findings highlighted three key insights. Firstly, high intra- and inter-rater reliability for pre- and postoperative VKA and VBH_A values could be achieved using both software platforms, with ICC values > 0.85° and SEMs <2.5°. These results support the routine clinical use of these parameters for both preoperative assessments and postoperative follow-up using radiography. Secondly, moderate to good reliability was observed for VBH_M measurements, especially in postoperative assessments, with ICC values from 0.75 to 0.85. This suggests greater observer-dependent variability, likely influenced by the challenge of accurately identifying medial vertebral landmarks in demineralized osteoporotic bone.

While both radiographic VKA and VBH measurements are valuable, VKA tends to be more reliable and shows greater agreement, particularly for assessing longitudinal changes after surgery. Thirdly, the measurement of radiographic changes over time (responsiveness) showed moderate reliability, particularly when using software #1 (Weasis). In contrast, software #2 (OsiriX MD) yielded higher ICC values and narrower SEM ranges, suggesting that it may be better suited to longitudinal studies or clinical follow-up.

These results have practical implications for the spine surgeons and radiologists involved in managing OVCFs (Goldstein and CNCTORD, 2015). VKA was the most robustly reliable metric across all raters and time points. Although VKA_II measurements appeared slightly more optimistic postoperatively, their susceptibility to discal compensation may confound assessments of true vertebral deformities and pain generation. In contrast, measuring VKA_SI provides a more consistent, clinically relevant evaluation. Indeed, this aligns with current clinical thinking and helps to avoid overestimating corrections due to the adjacent disc compensating by hyperextension, as previous literature has suggested (Schwab et al., 2006). In contrast, VBH-based parameters were slightly less reliable, especially VBH_M. This confirmed findings by Sadiqi et al. (2017), who reported moderate inter-rater reliability for vertebral body height ratios, emphasizing the difficulties of consistently identifying vertebral endplates between observers, even when standardized anatomical points are defined.

Regarding software tools, our inter-software agreement analysis revealed that while measurement variability was not significantly influenced by surgical experience, notable discrepancies existed between the two software tools. Limits of agreement were narrower between senior surgeons, highlighting a learning curve effect and suggesting intrinsic software-related variations. For instance, OsiriX MD showed greater measurement consistency, especially in postoperative VKA and VBH_A assessments. These findings suggested that software architecture, image manipulation tools (e.g., magnification, edge enhancement), and user interface ergonomics can significantly affect measurement precision (Mokkink TCKDSPAJPD et al., 2010).

OsiriX MD software's consistent outperformance of Weasis, in terms of intra- and inter-rater agreements for changes after surgery, supports its preferential use in situations where longitudinal precision is paramount, such as in clinical trials, follow-up evaluations, and surgical outcome monitoring. Given that no previous studies, to the best of our knowledge, have directly compared software-specific reliability in this context, our findings contribute novel and clinically actionable evidence to this area of spine measurement. This supports the generalizability of the findings across commonly used DICOM viewers.

Responsiveness (the ability to detect post-operative change) was only moderate for both VKA and VBH, indicating that quantifying post-operative changes remains challenging, especially for vertebral height, which is affected by endplate irregularity and radiographic projection variability in demineralized bone (Alanay et al., 2007).

This study had several strengths. Firstly, it addressed a highly prevalent and clinically significant condition in aging populations—OVCFs—where accurate radiological monitoring is critical for informed therapeutic decision-making (Panda and DCBU, 2014). Secondly, its design was methodologically rigorous, incorporating a pre-calculated sample size based on ICC, a blinding of raters, and a random selection of participants stratified by OF classification. Importantly, including multiple raters with varying levels of clinical expertise (orthopedic surgery residents, senior spine surgeons, and one non-orthopedic surgeon) enhanced the study's generalizability and the replicability of findings. Thirdly, the assessment of both intra- and inter-rater reliability, as well as of software agreement and responsiveness to surgical change, provided a holistic evaluation of measurement quality, responding directly to the core measurement property standards defined by Mokkink et al. (Mokkink TCKDSPAJPD et al., 2010; Terwee PJCRLJTBCD et al., 2021). The use of both ICC and SEM metrics, as well as Bland–Altman plots for visualizing agreement, ensured the study's statistical robustness as per the recommendations in the COnsensus-based Standards for the selection of health status Measurement Instruments (COSMIN) (Mokkink TCKDSPAJPD et al., 2010).

### 4.1 Limitations

Nevertheless, the study had some limitations. The sample size, while sufficiently powered for an ICC analysis, was modest and drawn from a single institution, potentially limiting the study's broader applicability. Additionally, only patients who had undergone surgical treatment were included, which may have introduced a selection bias toward more severe cases of OVCF. Non-surgical cases may have exhibited different patterns of deformity or radiographic change, which could have affected our reliability metrics. Another limitation was the relatively short postoperative follow-up period (2–5 days). This may not have fully captured long-term measurement variability or hardware-related complications (e.g. progressive collapse or cement subsidence), which could have influenced the identification of vertebral landmarks. Moreover, while the raters were blinded to previous measurements, complete blinding to the clinical context or treatment protocols used was not feasible. Concerning software, we decided to compare the two platforms available within our institution and most commonly used in our clinical practice. Finally, our study concentrated on 2D imaging, which is routinely used during the diagnostic and follow-up phases. This choice was based on the significance of the progression of deformities under the influence of gravity. However, it is important to note that 3D imaging techniques, such as CT scanning and MRI, are generally necessary for a comprehensive assessment of fracture morphology (Al et al., 2024).

### 4.2 Perspectives

To reach more definitive conclusions, future studies should aim to expand the comparative assessment of radiographic measurement tools across more platforms, ideally in multicenter settings.

The potential for artificial intelligence (AI) or semi-automated software to standardize vertebral landmark identification also warrants investigation. AI-powered tools are likely to become increasingly accessible, supporting radiologists, spine surgeons, and general practitioners alike. This democratization of diagnostic technology should enable more reliable and reproducible monitoring of OVCFs by the different specialties. Such integration could lead to earlier, simpler interventions rather than more complex, delayed surgeries, thus reducing costs and improving patient outcomes.

From a clinical perspective, developing standardized protocols that prioritize the measurement of the VKA_SI and the anterior VBH—the most reliable parameters—could reduce inter-clinician disagreements and improve treatment consistency, especially in multidisciplinary teams.

## 5 Conclusion

The present study confirmed that the VKA and VBH were the most reliable and reproducible measurements to analyze when assessing vertebral deformities in patients with osteoporotic vertebral compression fractures. The VKA demonstrated the highest intra- and inter-rater reliability, especially when evaluating change after surgery. Among the two software platforms examined, OsiriX MD demonstrated greater consistency, suggesting its suitability for longitudinal assessments. Although both the VKA and VBH are viable measurements in clinical practice, the former should be favored when therapeutic decisions hinge on radiological progression or correction. The present findings should help inform initiatives to standardize spinal trauma assessment and encourage the further development of precision imaging tools.

## Contributor's statement

Dr. Tsoupras designed the study, did the data acquisition, wrote and revised the initial manuscript until a final version was achieved.

Dr. Tabard-Fougère designed the study, did the data acquisition, did the statistical analysis, created the graphics for the study, wrote and revised the initial manuscript until a final version was achieved.

Dr. Al Taha participate to the study design, did the data acquisition, validated the results, critically reviewed the manuscript, and made important contributions to the final version.

Dr. Feltri and Dr. Boudabbous critically reviewed the manuscript and made important contributions to the final version.

Dr. Dominguez and Dr. Lauper designed the study, supervised the project, did the data acquisition, critically reviewed the manuscript, and made important contributions to the final version.

All the authors approved the final manuscript as submitted and agree to be held accountable for all aspects of the work.

## Funding

None.

## Declaration of competing interest

The authors declare that they have no known competing financial interests or personal relationships that could have appeared to influence the work reported in this paper.
